# Green Synthesis of Molecularly Imprinted Polymers: Advances Toward Sustainable Materials

**DOI:** 10.3390/polym18040512

**Published:** 2026-02-19

**Authors:** Alessandra Cutaia, Giancarla Alberti

**Affiliations:** Department of Chemistry, University of Pavia, Via Taramelli 12, 27100 Pavia, Italy; alessandra.cutaia01@universitadipavia.it

**Keywords:** molecularly imprinted polymers, green synthesis, bio-based monomers, sustainable materials

## Abstract

Molecularly imprinted polymers (MIPs) are synthetic materials with highly selective recognition properties and are widely studied for applications in separation, sensing, catalysis, and biomedical analysis. However, conventional MIP synthesis often relies on toxic solvents and reagents, causing environmental and sustainability concerns. This review critically examines recent advances in the green synthesis of MIPs, focusing on strategies aligned with green chemistry principles. Emphasis is placed on the use of environmentally less toxic solvents, as well as bio-based and less hazardous functional monomers and crosslinkers. Emerging polymerization techniques, such as microwave-assisted, photochemical, and solvent-free approaches, are also discussed. The impact of green synthetic routes on the structural, physicochemical, and recognition properties of MIPs is analyzed, highlighting both benefits and current limitations. Finally, key challenges and future perspectives for the development of sustainable MIPs are outlined.

## 1. Introduction

In recent decades, chemistry-related matters have experienced rapid growth due to their central role in modern technological development. In particular, the design and synthesis of advanced functional materials have attracted considerable attention, as synthetic strategies and precise control over structural features strongly affect their performance [1]. However, conventional synthetic routes typically rely on large amounts of organic solvents, excess reagents, and energy-intensive procedures, raising concerns about sustainability [2].

Moreover, the large-scale production and commercialization of advanced materials have intensified the use of nonrenewable and hazardous chemicals, resulting in significant environmental and health challenges [3,4]. Although such materials are indispensable in areas including sensing, medicine, electronics, and catalysis, their synthesis and use often conflict with the sustainability principles [5,6,7]. The long-term ecological consequences of these practices are expected to persist, encouraging the scientific community to implement strategies to mitigate the adverse impacts of chemical research and industrial activities [8,9,10].

In response to these challenges, green chemistry has emerged as a guiding framework across multiple disciplines, including synthesis, analytical chemistry, engineering, and pharmaceutical sciences [11,12,13,14]. Growing environmental awareness within society has accelerated the adoption of sustainable practices, supported by regulatory agencies, funding bodies, and dedicated scientific journals. As a result, green chemistry has become a defining paradigm of 21st-century research, with substantial global investment directed toward reducing the environmental footprint of chemical technologies [15,16].

Among advanced functional materials, MIPs are widely recognized as highly selective, tailor-made polymeric systems that contain recognition sites complementary in size, shape, and chemical functionality to specific target molecules [17].

**Figure 1:**
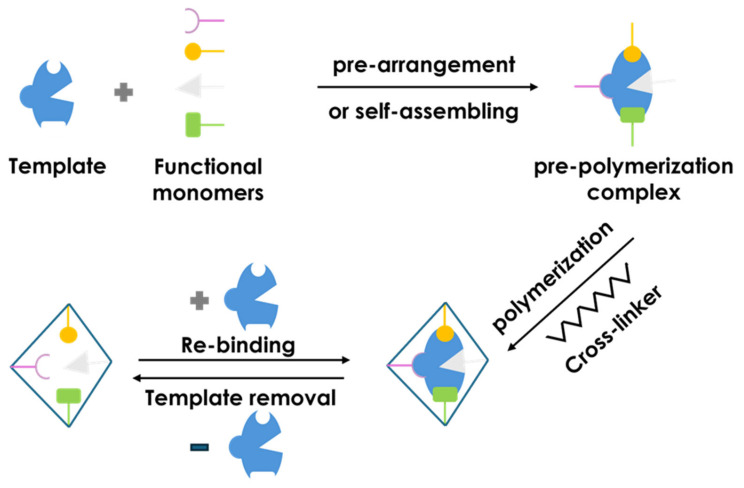
General scheme of MIP synthesis.

The synthesis of MIPs generally involves three main stages, as schematized in Figure 1. First, a pre-polymerization complex forms via self-assembly or specific non-covalent interactions between the template and functional monomers. This is followed by the addition of cross-linkers and initiators and subsequent thermal or photo-induced polymerization, which fixes the spatial arrangement of the template–monomer complex within a rigid polymeric network. Finally, removal of the template using suitable solvent systems generates permanent binding cavities that retain a “molecular memory” of the target, enabling selective recognition and rebinding [18,19].

Despite the successful commercialization and widespread application of MIPs, their environmental and health impacts remain insufficiently addressed. MIP synthesis has traditionally emphasized performance and selectivity, often neglecting the risks associated with toxic reagents, solvent-intensive processes, and waste generation [20,21]. In addition to potential operator exposure during synthesis, the high chemical stability and poor degradability of MIPs raise concerns regarding their persistence, accumulation, and possible ecological toxicity after disposal, with waste streams from both imprinted and non-imprinted polymers representing an additional environmental burden [21]. Consequently, the integration of green chemistry principles into molecular imprinting has become increasingly important. However, despite broad recognition of this need, practical implementation remains limited due to the lack of imprinting-specific sustainability guidelines. While general green chemistry principles provide a useful foundation, they are not fully suited to the unique features of molecular imprinting, prompting the recent proposal of tailored sustainability principles to guide the development of greener MIP synthesis strategies [21,22].

The present review is structured into three main sections: the first focuses on the application of green reagents in MIP synthesis; the second analyzes more sustainable approaches to synthetic pathways; the third identifies devices/instruments that can be used as tools to achieve greener and more sustainable goals.

## 2. MIP Synthesis Exploiting Green Reagents

As highlighted above, the synthesis of MIPs has traditionally relied on petrochemical-derived monomers, toxic solvents, and energy-intensive processes; in response, significant efforts have been devoted to redesigning MIP synthesis in line with green chemistry principles.

This section provides a critical overview of recent strategies for exploiting environmentally friendly reagents in MIP synthesis, focusing on replacing or modifying conventional functional monomers, crosslinkers, initiators, porogenic solvents, and templates. Special attention is given to ionic liquids, deep eutectic solvents, bio-based polymers, and alternative imprinting strategies, highlighting both their potential advantages and the limitations that must be carefully addressed to achieve sustainable and high-performance MIPs.

### 2.1. Functional Monomers

MIP synthesis typically begins with the formation of a pre-polymerization complex between the template molecule and suitable functional monomers, whose chemical nature largely determines the selectivity and binding performance of the resulting polymer. Historically, a wide range of vinyl-based monomers has been employed for this purpose, including methacrylic acid (MAA), acrylamide (AAM), methacrylamide, and N-vinylpyrrolidone [23,24]. Among these, acrylic-based monomers have been widely used because they form strong noncovalent interactions (e.g., hydrogen bonding and electrostatic interactions) with a wide range of template molecules, yielding MIPs with high binding affinity and versatility.

Despite their widespread use, acrylic-derived monomers raise significant environmental and toxicological concerns. Several of these compounds are classified as hazardous to freshwater and marine organisms and are commonly used in combination with volatile, toxic organic solvents [25]. From the perspectives of green analytical chemistry (GAC) and, more broadly, green chemistry, the extensive use of such monomers is increasingly questioned. Their minimization or substitution with less hazardous, more sustainable alternatives has become a key objective in developing greener MIP synthesis routes.

In this scenario, ionic liquids (ILs) have attracted considerable attention. ILs are molten salts with melting points near room temperature and were initially proposed as green materials because of their tunable properties, negligible vapor pressure, and non-flammability [26,27].

Their ability to form multiple interactions with organic molecules—including hydrogen bonding, ion-exchange, electrostatic, hydrophobic, and π–π interactions—has promoted their use not only as solvents but also as functional monomers or monomeric components in MIP synthesis, thereby enhancing template–monomer recognition [28].

Representative examples include vinyl- or allyl-functionalized imidazolium-based ILs, such as 1-vinyl-3-butylimidazolium chloride, 1-vinyl-3-methylimidazolium chloride, and 1-allyl-3-methylimidazolium bromide, which have been successfully employed as functional monomers in MIP synthesis [29,30,31].

The interest in IL-based MIPs is driven primarily by green-chemistry properties, such as low volatility, high thermal stability, high ionic conductivity, and compatibility with a broad range of organic solvents. Nevertheless, growing evidence has called into question their actual environmental sustainability. Several ILs exhibit significant toxicity toward aquatic organisms and poor biodegradability, while the toxicological profiles of many compounds remain incomplete. In addition, their high water solubility may facilitate environmental dispersion, indicating that ILs should no longer be considered intrinsically “green” and that their use in MIP synthesis requires careful, case-specific evaluation [32,33].

In parallel, increasing attention has been devoted to functional monomers derived from renewable and bio-based resources, particularly those obtained from biomass waste [34,35]. These materials aim to further reduce environmental impact while preserving or enhancing molecular recognition performance. In this context, naturally occurring polymers and macrocyclic compounds, such as chitosan and cyclodextrins, have emerged as promising functional components for MIP synthesis owing to their structural versatility and intrinsic affinity toward a wide range of target molecules [36].

Chitosan, a naturally occurring polysaccharide obtained by the deacetylation of chitin, is among the most extensively investigated bio-based materials for the development of sustainable MIPs. Its abundance, renewability, low cost, and derivation from biomass waste (e.g., crustacean shells) make chitosan particularly attractive from both environmental and economic perspectives. From a chemical standpoint, chitosan exhibits a high density of primary amine and hydroxyl functional groups, which enable multiple non-covalent interactions with template molecules, including hydrogen bonding and electrostatic interactions [37].

Notably, these functional groups enable strong template–monomer interactions even in aqueous media, representing a significant advantage over conventional (meth)acrylic-based MIPs, which often require organic solvents for effective molecular recognition. As a result, chitosan-based MIPs typically exhibit enhanced biocompatibility and reduced toxicity, making them well-suited for applications in bioanalysis, environmental monitoring, and biomedical research.

Despite these advantages, the use of chitosan in molecular imprinting is not without limitations. Its poor solubility at neutral and alkaline pH, together with its intrinsic structural heterogeneity, may negatively affect imprinting efficiency and batch-to-batch reproducibility. To address these issues, chemical modification and crosslinking strategies are commonly employed; however, such approaches may partially compromise the overall greenness of the synthesis and therefore require careful optimization [38].

Cyclodextrins (CDs) are cyclic oligosaccharides obtained from starch by enzymatic degradation and are widely regarded as bio-based, biodegradable, and non-toxic materials. The most common native forms (α-, β-, and γ-CD) consist of six to eight α-D-glucopyranose units and exhibit a characteristic toroidal structure with a hydrophobic inner cavity and a hydrophilic outer surface, enabling host–guest inclusion complex formation with a wide range of organic molecules [39].

This supramolecular recognition mechanism has promoted the use of CDs as functional monomers or recognition elements in MIP synthesis, particularly for hydrophobic and aromatic templates. In contrast to conventional acrylic-based systems, CD-based MIPs rely primarily on inclusion interactions rather than classical hydrogen bonding, thereby enabling effective imprinting in aqueous media and aligning more closely with green chemistry principles. Moreover, the presence of multiple hydroxyl groups on the cyclodextrin rim facilitates chemical functionalization and polymer integration.

Nevertheless, the relatively large molecular size and rigid cavities of CDs may influence polymer morphology and limit applicability to size-compatible templates. Careful optimization of polymerization parameters is therefore required, and strategies based on covalent attachment of CDs to polymerizable units are often employed to improve control, albeit at the cost of increased synthetic complexity [40]. A distinctive advantage of CDs is their strong chiral discrimination, making CD-based MIPs particularly promising for enantioselective recognition and separation, especially in pharmaceutical applications [41].

Among bio-based and green functional monomers, dopamine has emerged as a versatile and sustainable building block for MIP synthesis. As a naturally inspired small molecule, dopamine can undergo spontaneous oxidative polymerization under mild alkaline and aqueous conditions, leading to the formation of polydopamine (PDA) without the need for external initiators, high temperatures, or toxic organic solvents. These features make dopamine particularly attractive within the framework of green chemistry [42,43].

The resulting PDA is a cohesive and strongly adhesive material, inspired by mussel adhesive proteins [44], and is characterized by a high density of catechol and amine functional groups. This chemical richness enables multiple non-covalent interactions with template molecules, including hydrogen bonding, π–π stacking, and metal coordination. Consequently, dopamine can act not only as a functional monomer but also, in some cases, as a crosslinker, contributing to the formation of stable, robust imprinted networks. In addition, the universal adhesive properties of PDA enable uniform coating on a wide variety of substrates, including metals, polymers, oxides, and carbon-based materials [45].

These characteristics have promoted the development of mussel-inspired surface imprinting strategies, in which thin MIP layers are directly deposited onto transducer surfaces or nanomaterials. Surface-confined PDA-based MIPs benefit from reduced diffusion distances, enhanced binding kinetics, and improved accessibility of recognition sites, making them particularly suitable for sensing and bioanalytical applications.

Despite these advantages, dopamine-based MIPs also present some limitations. The intrinsic heterogeneity of PDA and the limited control over film thickness and crosslinking density can negatively affect reproducibility and binding-site uniformity. To address these issues, electropolymerization of dopamine has been proposed as an effective alternative. In this approach, dopamine monomers are electrooxidized at the electrode surface, enabling precise control of film thickness and polymer growth via electrochemical parameters [46,47]. Electrosynthesized PDA-MIPs offer improved reproducibility, greater accessibility of imprinted sites, and straightforward integration onto electrode surfaces, further reinforcing their suitability for sustainable, high-performance sensing platforms [48].

Table 1 presents examples of MIPs prepared using green functional monomers.

**Table 1:** Examples of MIPs prepared using green functional monomers.

Template	Monomer	Sample Matrix	Analytical Technique	LOD	Ref.
propionamide	[H-DMAEMA][Acr]	-	UHPLC/DAD	-	[49]
hemoglobin	1-vinyl-3-propyl imidazole sulfonate	bovine blood	DPV	5.22 × 10^−15^ mg/mL	[50]
gallic acid	chitosan	red wine, red grape juice and plum juice	EW-OFCS	23 ng/mL	[51]
ketorolac	chitosan	human plasma	UV spectroscopy	0.08 μg/mL	[52]
Triclosan, hexachlorophene and 2,4-dichlorophenol	β-cyclodextrin	river water	HPLC	0.30 μg/L	[53]
Chloramphenicol, florfenicol, thiamphenicol	β-cyclodextrin	honey, milk, chicken, and pork	HPLC-UV	5–10 μg/L	[54]
Pyocyanin	Dopamine	spiked bacterial culture	SWV	0.74 μM	[46]
MDPEA	Dopamine	urine	DPV	37 nM	[47]

[H-DMAEMA][Acr]: 2-(Dimethylammonium) ethyl methacrylate acrylate; UHPLC/DAD: ultra-high performance liquid chromatography/diode array detector; DPV: Differential Pulse Voltammetry; EW-OFCS: Evanescent wave-Optical fiber chemical sensor; SWV: Square wave voltammetry; MDPEA: 3,4-methylenedioxyphenethylamine.

### 2.2. Cross-Linkers and Initiators

Crosslinkers play a crucial role in MIP synthesis, as they are responsible for the formation of the three-dimensional polymeric network surrounding the template molecules and stabilizing the imprinted cavities after template removal. The nature and amount of crosslinker strongly influence the mechanical stability, porosity, and binding performance of the resulting MIPs. Among the most commonly employed crosslinkers in conventional MIP formulations are divinylbenzene (DVB) and ethylene glycol dimethacrylate (EGDMA), owing to their high reactivity, commercial availability, and ability to form rigid, mechanically stable polymer networks.

Beyond conventional vinyl-based crosslinkers, ionic liquids (ILs) and deep eutectic solvents (DESs) have recently been explored as multifunctional crosslinking agents in MIP synthesis. In these systems, the crosslinker can simultaneously contribute to network formation and molecular recognition.

When DESs are used as crosslinkers, their components must bear polymerizable terminal groups to enable incorporation into the polymer network. Vinyl-functionalized compounds, such as acrylamide and vinyl ammonium salts (e.g., (3-acrylamidopropyl)trimethylammonium chloride), are therefore commonly used to prepare polymerizable DESs, enabling the formation of stable networks while providing multiple interaction sites for template recognition.

Similarly, room-temperature ionic liquids (RTILs) have been investigated as crosslinkers that can provide multiple modes of interaction with template molecules. However, the high density of interaction sites in RTIL-based systems may not be fully exploited during imprinting, thereby limiting their effectiveness as crosslinkers [55].

Polymerization is typically initiated by small amounts of radical initiators, which activate the polymerization process. 2,2′-Azobisisobutyronitrile (AIBN) is by far the most widely used initiator in MIP synthesis due to its thermal stability and reliable radical generation under conventional polymerization conditions.

Despite its widespread use, AIBN cannot be considered a green initiator because it is petroleum-derived, requires thermal activation at relatively high temperatures, and increases overall energy consumption during polymerization. From a sustainability perspective, these aspects represent clear limitations, particularly when thermally sensitive templates or bio-based components are involved.

In recent years, efforts have focused on developing and adopting alternative initiation strategies with reduced environmental impact. Photo-initiated polymerization, for instance, enables radical generation at room temperature or below under controlled light irradiation, significantly lowering energy input while preserving efficient template–monomer complexation [56].

Moreover, several low-energy or initiator-free polymerization approaches, including electropolymerization, microwave-assisted polymerization, and spontaneous oxidative polymerization (e.g., dopamine to polydopamine), completely eliminate the need for conventional radical initiators. These methods not only reduce chemical inputs but also offer enhanced control over polymer growth and morphology, aligning more closely with the principles of green chemistry [57].

Overall, while AIBN remains a benchmark initiator due to its robustness and reproducibility, its partial or complete replacement by greener initiators or initiator-free strategies represents an important step toward more sustainable MIP synthesis.

### 2.3. Porogenic Solvents

Porogenic solvents play a key role in MIP synthesis by promoting effective template–monomer interactions in the pre-polymerization mixture and by controlling the formation of the porous polymer network. The choice of porogenic solvent directly influences pore size, surface area, and morphology, all of which are crucial for analyte diffusion and accessibility to imprinted binding sites.

Conventional low-dielectric organic solvents, such as toluene and dichloromethane, have been widely used as porogens because they stabilize hydrogen-bonding and electrostatic interactions. However, their volatility, toxicity, and environmental persistence have raised increasing concerns, motivating the search for greener alternatives. Water represents the most environmentally benign option, particularly for hydrophilic and bio-based monomers, although its high polarity may weaken non-covalent interactions in classical imprinting systems.

Among alternative porogens, ionic liquids (ILs) and deep eutectic solvents (DESs) have attracted growing interest due to their low volatility and tunable physicochemical properties [58].

Imidazolium-based room-temperature ILs are the most commonly employed and have been shown to improve MIP performance under polar conditions and to enhance morphological stability by reducing polymer shrinkage or swelling. These features make ILs particularly attractive for applications involving aqueous or highly polar samples, although their use must be balanced against the toxicity and environmental concerns discussed previously [59].

DESs, typically composed of a hydrogen-bond acceptor (HBA) and a hydrogen-bond donor (HBD), provide a modular and potentially sustainable solvent platform. Choline chloride is frequently used as the acceptor due to its low toxicity and biodegradability [60]. Nevertheless, the use of DESs as porogens in MIP synthesis remains relatively limited because strong interactions with monomers and templates can interfere with the formation of well-defined recognition sites, and the mechanisms underlying these effects are incompletely understood [61].

Supercritical carbon dioxide (scCO_2_) represents another green alternative to conventional organic porogens. Above its critical point (31.1 °C, 73.8 bar), scCO_2_ combines high diffusivity with good solvating power for many commonly used monomers, while being non-toxic, non-flammable, and inert. However, broader implementation of MIP synthesis remains constrained by the complexity of high-pressure equipment, long processing times, and associated costs [62].

Although ionic liquids and deep eutectic solvents are often proposed as green alternatives, their use is associated with several non-negligible limitations that must be carefully evaluated. Ionic liquids, in particular, may suffer from high cost, elevated viscosity, and limited biodegradability, factors that can adversely affect mass transfer, template diffusion, and the morphology of the resulting polymers. Moreover, as discussed above, the environmental impact and long-term toxicity of many IL families remain insufficiently understood, raising concerns about their large-scale or routine application. Consequently, ILs should not be considered inherently green solvents but rather tunable functional media whose sustainability depends strongly on their chemical structure, concentration, and specific application context.

### 2.4. Templates

The template molecule plays a central role in molecular imprinting, as it defines the shape, size, and chemical functionality of the recognition cavities formed within the polymer matrix. A wide variety of compounds can be used as templates, including drugs, amino acids, hormones, pesticides, environmental pollutants, and, in more specialized applications, biomacromolecules such as proteins and microorganisms.

Template–monomer interactions, typically governed by non-covalent forces (e.g., hydrogen bonding, electrostatic and hydrophobic interactions), are essential for establishing an effective imprinted network. Accordingly, careful optimization of the template-to-monomer ratio is required to ensure the formation of stable and selective binding sites.

In the development of MIP-based analytical methods, a non-imprinted polymer (NIP) is routinely synthesized under identical conditions but in the absence of the template molecule. A comparison between MIPs and NIPs is crucial for demonstrating the presence of specific imprinted cavities and distinguishing actual imprinting effects from nonspecific adsorption. In this context, Baggiani [63] and co-workers systematically investigated a large set of polymers and demonstrated that imprinting does not generate entirely new recognition properties but rather enhances the polymer matrix’s pre-existing affinity for the target molecule. According to their findings, when a NIP already exhibits some affinity for the target, the corresponding MIP shows excellent recognition performance, whereas poor affinity in the NIP generally leads to weak imprinting effects. This concept, subsequently confirmed by other studies, highlights the importance of rational polymer design beyond the mere presence of a template.

From a safety and sustainability perspective, the use of certain template molecules raises significant concerns. During both the pre-polymerization complex formation and template removal steps, operators and the environment may be exposed to hazardous or toxic compounds. Therefore, template molecules should ideally be soluble in the reaction medium, stable under polymerization conditions, inexpensive, and, most importantly, non-toxic.

To address these issues, the use of dummy templates—structurally similar but less hazardous analogues of the target analyte—has gained increasing attention as a greener and safer alternative to direct imprinting with toxic analytes [64,65].

More advanced imprinting strategies have also been proposed to overcome limitations associated with template availability, toxicity, or molecular size. In fragment (or segment) imprinting, a representative portion of the target molecule, containing key functional groups, is used as a template; this approach is widely applied in biomolecular and microbial recognition [66,67].

Additionally, multi-template imprinting has been explored to generate MIPs that recognize families of structurally related compounds using a single polymer [68,69]. While promising, this approach may yield heterogeneous binding sites and synergistic or antagonistic effects among templates, potentially complicating recognition behavior in complex samples.

Overall, dummy-template, fragment-imprinting, and multi-template strategies expand the applicability of MIPs while reducing safety risks, template leakage, and environmental impact. However, their implementation requires careful design to balance selectivity, reproducibility, and sustainability.

## 3. Green and Sustainable Approaches for MIP Synthesis and Template Removal

In contrast to conventional approaches for the preparation of MIPs, emerging methodologies intrinsically incorporate principles of green chemistry.

Materials research is commonly structured around four complementary paradigms: experimental investigation, theoretical analysis, computational modeling and simulation, and data-driven methodologies [70]. Within this framework, classical computational techniques and machine-learning approaches are first outlined, emphasizing how computer-assisted strategies can markedly reduce the generation of unwanted by-products typically associated with empirical trial-and-error procedures [55]. Subsequently, in the context of the sequential experimental stages of MIP synthesis, state-of-the-art sustainable synthesis strategies will be discussed.

### 3.1. Computational Approaches

The development of smart molecularly imprinted materials with enhanced performance requires a thorough understanding of the underlying molecular interactions. Identifying appropriate polymer architectures typically requires extensive experimental work that is often labor-intensive, time-consuming, and resource-demanding in terms of reagents, solvents, and workforce. This process normally involves evaluating numerous substrate–polymer combinations and performing multiple experimental steps to achieve the desired material properties. The application of molecular modeling approaches can substantially alleviate these limitations. Computational simulations enable quantitative assessment of interactions among molecular components and the identification of optimal chemical arrangements before and during the synthesis of MIPs.

The conventional synthesis of MIPs largely relies on trial-and-error experimentation, which is time-consuming and resource-intensive. Advances in computational science have enabled the rational design of MIPs before laboratory synthesis, primarily through theoretical simulations to select suitable functional monomers, template molecules, and crosslinkers, and to determine their optimal ratios [71].

**Figure 2:**
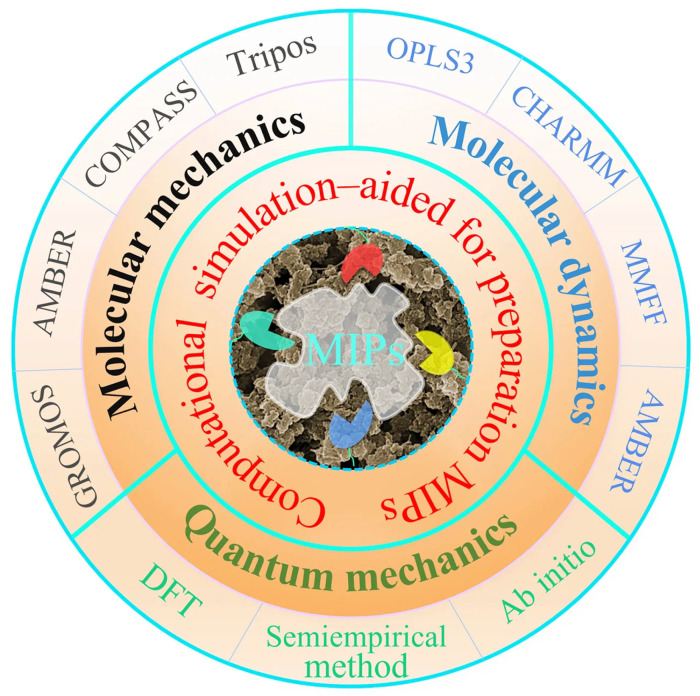
Traditional methods of computational simulation for MIPs. Reprinted with permission of [71]. Copyright 2021, MDPI.

Commonly employed simulation approaches include quantum mechanics (QM), molecular mechanics (MM), and molecular dynamics (MD) (see Figure 2), each offering distinct advantages in terms of accuracy and computational cost [55].

QM methods, particularly density functional theory (DFT), provide highly accurate descriptions of molecular interactions and are well suited to evaluating template–monomer binding energies, although at a high computational cost. In contrast, MM and MD treat atoms as the fundamental units, allowing more efficient simulations of larger systems. Among them, MD is especially valuable for capturing atomic motion and realistically modeling the imprinting and polymerization processes. In practice, these methods are often combined to exploit complementary strengths and to improve predictive reliability [71,72,73,74,75].

The theoretical design of MIPs generally involves identifying the most suitable functional monomer, optimizing component ratios, selecting crosslinkers and porogens, constructing the polymer structure, and evaluating predicted performance. These steps can now be accomplished mainly in silico, with stable template–monomer complex formation recognized as a key determinant of MIP selectivity and stability. Computational predictions have been shown to correlate well with experimental outcomes, thereby enabling significant reductions in material waste and costs, consistent with green chemistry principles [70,75].

In parallel, machine learning (ML) has emerged as a powerful data-driven approach for MIP design. Unlike traditional simulation methods, ML directly extracts structure–property relationships from experimental or computational datasets to predict material performance. Recent studies have demonstrated the potential of ML models to estimate MIP binding affinities and accelerate functional monomer screening [71,76,77]. However, model accuracy is strongly dependent on the quality and diversity of the training data, underscoring the importance of reliable datasets [55,78].

Overall, both physics-based simulations and ML approaches offer promising pathways toward efficient, sustainable MIP design. While experimental validation remains essential, the integration of computational modeling, data-driven methods, and green synthesis strategies is expected to play a central role in the future development and industrial translation of MIPs.

### 3.2. MIP Synthesis: Towards the Use of Green Strategies

Bulk, precipitation, and suspension polymerization are among the most commonly employed methods for synthesizing MIPs. However, each presents limitations when evaluated from a green chemistry standpoint.

In bulk polymerization, the reaction yields a solid monolith that must be crushed and sieved to achieve the desired particle size. This step is labor-intensive, time-consuming, and may pose health risks due to dust generation. Furthermore, approximately half of the resulting material is classified as solid waste during processing. Moreover, both precipitation and suspension polymerization require substantial quantities of organic solvents (porogens), thereby generating significant volumes of liquid waste [79].

In developing innovative imprinting strategies, the safety of operators and the environment should be prioritized by integrating green chemistry and molecular imprinting technology (MIT).

In the following paragraphs, some of the most environmentally friendly methods recently proposed will be discussed.

#### 3.2.1. Aqueous Precipitation Polymerization

Aqueous precipitation polymerization (APP) is a sustainable strategy for the synthesis of MIPs that aligns well with green chemistry principles [22]. Originally introduced by Shea and co-workers [80], this approach enables polymer formation in water under mild conditions, typically at ambient temperature. Early implementations employed acrylamide-based monomers, acrylic acid, bisacrylamide crosslinkers, peptide templates, and, in some cases, ionic surfactants. More environmentally benign variants have since been developed, in which surfactants are eliminated, and polymerization is initiated through photochemical or enzymatic routes [81,82].

**Figure 3:**
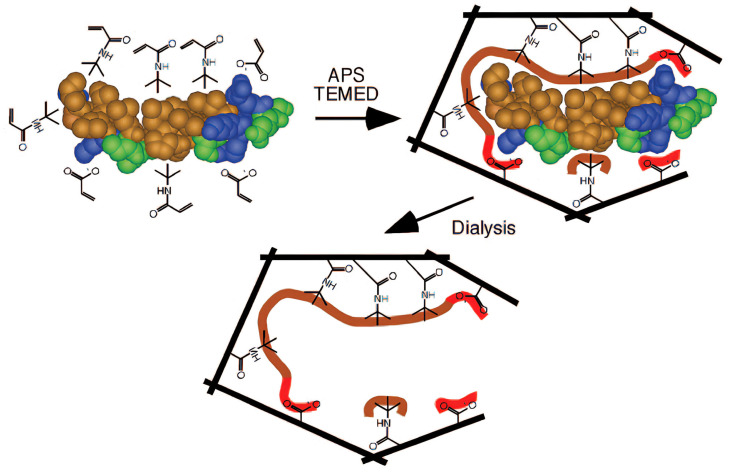
Schematic representation of the bee toxin melittin (Mel, a 26-amino acid peptide) imprinting process. Hydrophobic, positively or negatively charged, and hydrophilic residues are represented respectively in brown, blue, and green, respectively. Reprinted with permission of [80]. Copyright 2008, American Chemical Society.

The use of aqueous media and low-temperature conditions has enabled successful imprinting of a wide range of templates, ranging from small organic molecules to complex biomacromolecules, including enzymes, peptides, proteins, and antibodies [81,82,83,84,85]. For example, Figure 3 shows a schematic of peptide-imprinted polymer nanoparticle synthesis using the APP method.

APP is known to produce relatively homogeneous crosslinked polymer networks, resulting in improved binding performance and enhanced control over MIP surface properties. Nevertheless, the method is limited by the need for extensive post-synthesis purification steps, such as dialysis or column separation, which remain time-consuming and generate additional waste [22].

#### 3.2.2. Surface Imprinting

Conventional molecular imprinting approaches often suffer from inherent limitations, such as excessive template entrapment, incomplete template removal, limited adsorption capacity, slow mass transfer, and poor reusability of the resulting polymers.

Surface molecular imprinting technology, also referred to as carrier-assisted imprinting, has been developed to address these drawbacks. In this strategy, polymerization is confined to the surface of a solid support, yielding surface MIPs (SMIPs) [55].

Surface imprinting typically relies on techniques such as grafting, precipitation, or emulsion polymerization to form a thin imprinted layer on pre-functionalized carrier materials. As a result, recognition sites are predominantly located at or near the substrate surface, thereby enhancing accessibility, accelerating binding and release, and enabling more efficient template removal. Compared with bulk-imprinted polymers, in which templates are embedded within the polymer matrix, SMIPs require fewer washing steps and consume less solvent during template extraction [22,86]. These advantages make surface imprinting an attractive and environmentally friendly route for MIP fabrication.

Current surface imprinting strategies can be broadly classified into three main approaches: oriented surface imprinting, hollow or porous imprinting, and solid-phase synthesis.

(i) Oriented surface molecular imprinting technology (OMIT)

**Figure 4:**
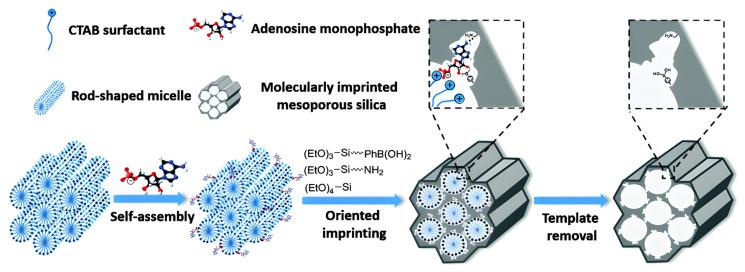
Schematic representation of dual-template docking-oriented molecular imprinting (DTD-OMI), for the preparation of AMP-imprinted MCM-41 mesoporous silica nanoparticles. Reprinted with permission of [88]. Copyright 2015, Royal Society of Chemistry.

Oriented surface molecular imprinting technology relies on specific interactions between functionalized solid supports and template molecules. In this approach, templates are immobilized on the carrier surface in a well-defined orientation, after which functional monomers and crosslinkers are polymerized to form an imprinted layer with controlled thickness. Subsequent solvent treatment removes the templates, leaving uniformly oriented recognition sites. A key advantage of OMIT is the high homogeneity of binding sites, which arises from the spatially controlled arrangement of template–monomer interactions. Unlike conventional imprinting methods, OMIT requires prior immobilization of template molecules on the substrate via tailored surface modification strategies [87]. For example, Figure 4 shows a schematic of a strategy, called dual-template docking-oriented molecular imprinting (DTD-OMI), for the preparation of AMP-imprinted MCM-41 mesoporous silica nanoparticles [88].

Boronate affinity-based imprinting is among the most commonly employed OMIT approaches, particularly for biosensor fabrication [89]. Sensors prepared using this strategy, such as quartz crystal microbalance (QCM) devices, have demonstrated markedly enhanced sensitivity and selectivity toward target proteins, achieving detection limits in the low ng/mL range [90]. Additional improvements have been reported by integrating oriented imprinting with surface modifications, such as PEGylation, thereby increasing adsorption capacity and selectivity in complex biological environments [87]. Furthermore, OMIT has been applied to the preparation of protein-imprinted fluorescent sensors, in which controlled orientation has led to significantly improved imprinting efficiency and detection sensitivity [91]. Despite these advantages, the requirement for template immobilization prior to polymerization adds complexity to the synthesis procedure, which may limit the scalability and industrial applicability of OMIT-based materials [55].

(ii) Hollow porous molecular imprinting polymers (HMIPs)

**Figure 5:**
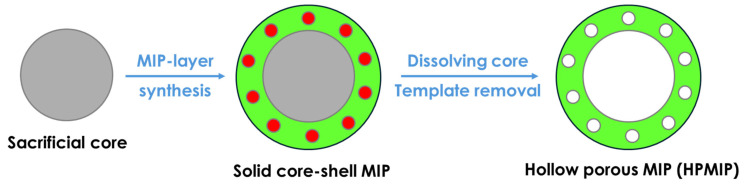
General scheme of the Hollow porous MIP (HPMIP) preparation.

Hollow porous MIPs (HMIPs) have attracted growing interest as advanced recognition materials, as they effectively address several limitations of conventional MIPs, including the restricted accessibility of binding sites, limited resolution, and slow mass transfer. Owing to their hollow and porous architecture, HMIPs exhibit a high surface-to-volume ratio, enhanced adsorption capacity, and improved transport kinetics for target molecules. HMIPs are typically prepared by forming an imprinted polymer shell around a sacrificial template or core, which is subsequently removed through physical or chemical treatments to generate internal recognition cavities (see the schematic of the procedure in Figure 5). In most cases, the core material contributes little to molecular recognition, if at all, relying instead on nonspecific interactions [92]. The presence of both inner and outer accessible surfaces provides HMIPs with a higher density of effective binding sites compared with bulk-imprinted polymers [93]. A variety of fabrication strategies have been developed for HMIPs, including sacrificial core templating, Pickering emulsion polymerization, seed swelling, imprinting on preformed hollow supports, and suspension polymerization. Recent studies have highlighted the strong potential of HMIPs as adsorbents in analytical and environmental applications, particularly for sample pretreatment and the selective extraction of trace-level analytes from complex matrices [94,95]. Their performance in removing environmental contaminants and improving analytical efficiency underscores their relevance in molecular imprinting technology. Despite these advantages, the hollow and porous nature of HMIPs often results in limited mechanical robustness. To prevent structural collapse, thicker imprinted shells are typically required, which can reduce mass-transfer efficiency and limit the effective utilization of recognition sites. Moreover, the use of sacrificial templates can increase material costs and environmental burden. Future research should therefore focus on enhancing the mechanical stability of HMIPs and developing more sustainable and economically viable fabrication strategies.

(iii) Solid-phase synthesis of MIPs

**Figure 6:**
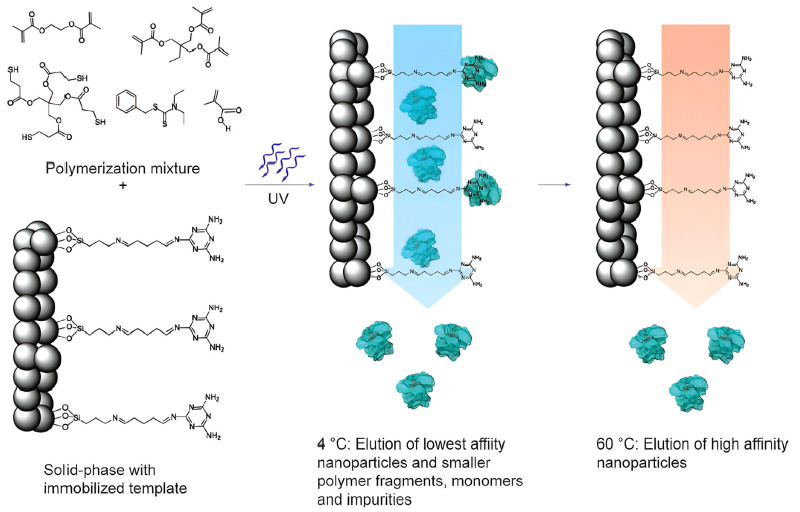
Scheme of the solid-phase synthesis of MIP nanoparticles. Reprinted with permission of [96]. Copyright 2013, John Wiley and Sons.

Solid-phase synthesis, first introduced in 2013 [96], represents an advanced extension of surface molecular imprinting that integrates key advantages of oriented and porous MIPs [97]. In this approach, template molecules are immobilized onto solid supports, such as functionalized glass beads, via affinity interactions rather than being dispersed freely in solution. The template-loaded supports are then introduced into the polymerization mixture under controlled conditions, thereby forming imprinted nanoparticles at the solid–liquid interface. These nanoparticles remain attached to the support until they are selectively released via affinity-based purification (see the procedure scheme in Figure 6).

Compared with conventional imprinting techniques, solid-phase synthesis is less labor-intensive and is not restricted to single-batch processing. The resulting MIPs typically display highly uniform binding characteristics, while the immobilized templates can be recovered and reused. In addition, the method is well-suited to automation and standardization, making it attractive from both sustainability and industrialization perspectives. Despite these advantages, solid-phase imprinting often exhibits relatively low product yields, as evidenced by a high environmental factor. Nevertheless, in conjunction with related surface imprinting approaches, such as chemical grafting and electropolymerization, solid-phase synthesis remains a promising strategy for the controlled and green fabrication of high-performance MIPs [98].

#### 3.2.3. Microcontact Imprinting

**Figure 7:**
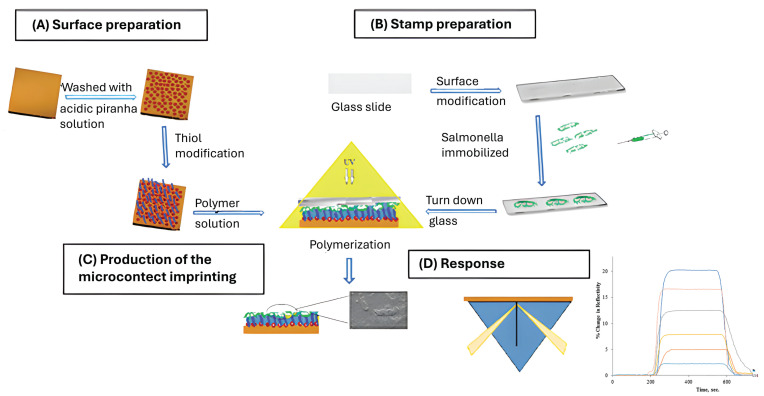
MIP-based plasmonic nanosensors for *Salmonella paratyphi* prepared by microcontact imprinting. (**A**) preparation of the surface plasmon resonance (SPR) chip surface; (**B**) *Salmonella. paratyphi* stamps preparation; (**C**) microcontact imprinting; (**D**) response graph of the SPR sensor. Reprinted with permission of [101]. Copyright 2017, MDPI.

Microcontact imprinting is a versatile and straightforward approach for generating well-defined, patterned surfaces, particularly suited for applications in biosensing, bioassays, tissue engineering, and controlled cell adhesion. The technique combines microcontact printing with molecular imprinting, enabling the transfer of template patterns—most commonly biomacromolecules—from a microstructured stamp onto a prepolymer or soft-polymer layer. Following contact, the polymer is cured, thereby embedding the templates at the interface between the stamp and the polymer matrix [99]. The rheological properties of the prepolymer play a critical role in determining imprinting quality. Materials with excessive rigidity may resist stamp deformation but can require high mechanical pressure, potentially damaging fragile biological templates. Conversely, overly soft polymers may lead to stamp adhesion or deformation of the imprinted features during demolding. When appropriately optimized, microcontact imprinting can be performed without solvents, making it one of the most environmentally benign imprinting strategies. This platform allows rapid, parallel fabrication of MIPs with diverse compositions using minimal amounts of monomer solution, which is particularly advantageous for rare, costly, or hazardous templates. In addition, template solubility constraints associated with conventional porogens are largely avoided. The formation of ultrathin imprinted layers minimizes template entrapment and yields highly uniform and accessible recognition sites [100]. A wide variety of organic and inorganic polymer systems, including conductive polymers, elastomers, thermoplastics, and sol–gel materials, have been successfully employed, many of which are commercially available, facilitating adoption by non-specialists. Figure 7 shows an example of MIP-based plasmonic nanosensors prepared by microcontact imprinting [101].

Despite these benefits, microcontact imprinting remains limited in scope. Its application to very soft or fragile biological entities is challenging, and achieving high selectivity for large, asymmetric templates through controlled orientation remains complex. Further methodological development is therefore required to expand the range of compatible templates and improve recognition performance [22].

### 3.3. Low-Energy MIP Synthesis

Beyond the choice of greener monomers, crosslinkers, solvents, and templates, the sustainability of molecular imprinting is strongly influenced by the energy input required during polymerization. Conventional thermal MIP synthesis typically relies on prolonged heating and radical initiators, resulting in high energy consumption and limited compatibility with thermally sensitive templates or bio-based components.

In this context, low-energy polymerization strategies have attracted increasing interest as practical tools to reduce the environmental footprint of MIP synthesis. Techniques such as electropolymerization, photo-induced polymerization, and microwave-assisted polymerization enable rapid polymer formation under mild conditions, often without the need for high temperatures or excessive amounts of chemical initiators. These approaches not only align with the principles of green chemistry but can also offer improved control over polymer architecture, morphology, and recognition-site accessibility.

#### 3.3.1. Electropolymerization

Electropolymerization for MIP synthesis is initiated by the electrochemical oxidation of an electroactive monomer in the presence of a template molecule within an electrochemical cell. Upon application of a controlled potential or current, monomer oxidation results in the growth of a polymer film directly on the working electrode surface, which serves as the transducer. Commonly employed electroactive monomers include pyrrole, thiophene, and dopamine, which can polymerize under mild conditions using amperometry or cyclic voltammetry [102,103].

One of the main advantages of electropolymerization lies in the precise control of film thickness, which can be readily tuned by adjusting the total charge passed at a constant potential or by varying the number of voltammetric cycles. This feature significantly enhances the reproducibility of the resulting electrosynthesized MIPs (eMIPs) compared to conventional bulk or solution-based polymerization methods. Additionally, electropolymerization is typically conducted at room temperature in aqueous or low-toxicity media, making it particularly suitable for thermally sensitive templates and bio-based components [104].

From a sustainability perspective, electropolymerization does not require external radical initiators and involves reduced consumption of organic solvents and toxic reagents. These characteristics, combined with intrinsic compatibility with sensor fabrication and surface-confined imprinting, position electropolymerization as an environmentally friendly and low-energy strategy for the preparation of MIPs [105].

Despite its numerous advantages, electropolymerization also presents some inherent limitations that may limit its broader application in MIP synthesis. First, the method is inherently limited to electroactive monomers, thereby significantly narrowing the range of functional monomers compared with conventional free-radical polymerization. This constraint may limit the diversity of chemical interactions available for template recognition.

Additionally, electropolymerization typically yields thin polymer films confined to electrode surfaces, which, while advantageous for sensing applications, hampers the scalability and bulk production of MIPs for applications such as solid-phase extraction or large-scale separation. The thickness of the electropolymerized layer must be carefully optimized, as excessively thick films can hinder mass transfer and reduce accessibility to imprinted binding sites, whereas overly thin films may compromise binding capacity.

**Figure 8:**
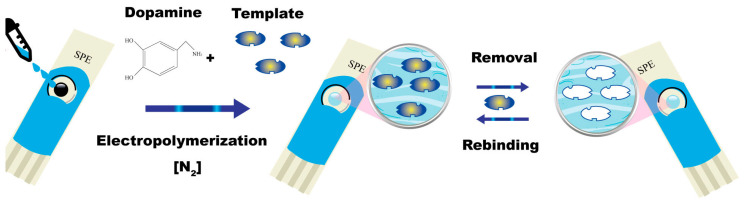
Electropolymerization was used to synthesize a polydopamine-based MIP, functionalizing the graphite working electrode of a screen-printed cell. Reprinted with permission of [48]. Copyright 2023, MDPI.

For example, Figure 8 presents a scheme for preparing an electropolymerized molecularly imprinted polydopamine sensor. In this case, the prepolymeric solution, containing the template and the functional monomer (dopamine), was drop-coated on the working electrode surface of a screen-printed cell. After the electropolymerization, the template removal can be performed electrochemically or by extraction with a suitable solvent or mixture of solvents [106].

#### 3.3.2. Photo-Induced Polymerization

Photo-induced polymerization has emerged as an attractive low-energy approach for MIP synthesis due to its rapid initiation kinetics and ability to operate under mild thermal conditions. Unlike conventional thermal polymerization, photopolymerization can be carried out at ambient or sub-ambient temperatures, which is particularly beneficial for preserving non-covalent interactions between the template and functional monomers during the pre-polymerization stage. Several studies have shown that lowering the polymerization temperature enhances the stability of the template–monomer complex, thereby improving imprinting efficiency and binding selectivity [107].

An important strength of photo-induced polymerization is its high degree of flexibility in terms of MIP format. This technique can be applied to the fabrication of monoliths, micro- and nanoparticles, surface-supported films, and other nanostructured materials. In combination with the wide range of commercially available photoinitiators, this versatility makes photopolymerization suitable for diverse imprinting strategies and application areas. Moreover, the polymerization process can be precisely controlled in space and time by modulating the irradiation area and light intensity, enabling fine regulation of polymer growth, crosslinking density, and microstructure along all three spatial dimensions [108].

**Figure 9:**
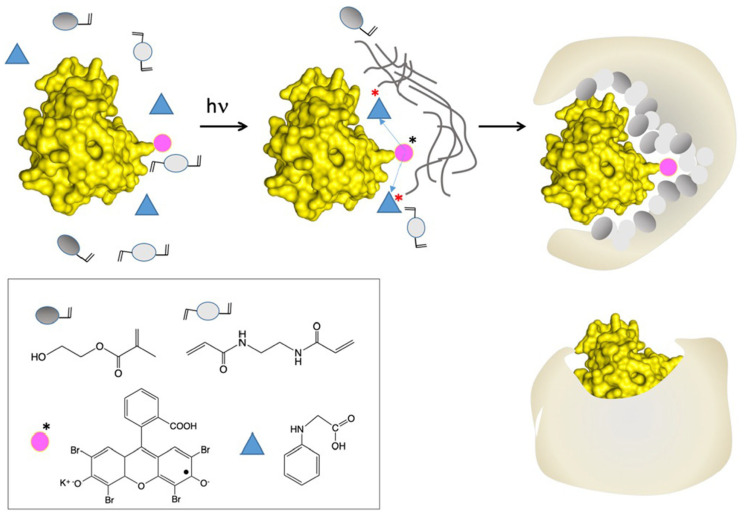
Photochemically initiating molecularly imprinted nanoparticles using protein templates. A template-bound macroinitiator is obtained by attaching a photoreactive dye, such as Eosin Y, to the target protein. This construct is then introduced into a diluted monomer mixture and exposed to light, which initiates polymer growth exclusively at the template site. As a result, polymer nanoparticles develop around single protein molecules. After formation, the molecularly imprinted nanoparticles can be detached from the protein and subsequently rebound, showing selective recognition of the specific protein they were designed to match. Asterisks in the figure represent radical species. Reprinted with permission of [110]. Copyright 2020, John Wiley and Sons.

An example of this strategy is reported in Figure 9.

However, several challenges limit the universal applicability of photopolymerization in MIP synthesis. One major drawback is the limited penetration of light into opaque or highly scattering reaction media, which can lead to non-uniform polymerization, particularly in thick or bulk samples. Variations in local light intensity may lead to heterogeneous polymerization rates, uneven phase separation, and the formation of materials with spatially varying porosity. While these effects can be mitigated by employing dilute systems, efficient mixing, or precipitation- and emulsion-based polymerization schemes, they remain problematic for large-scale monolithic MIPs. In addition, the exposure of the pre-polymerization mixture to UV or high-energy radiation may adversely affect light-sensitive templates, potentially causing chemical degradation or conformational changes that impair the fidelity of the imprinted binding sites [109].

Taken together, photo-induced polymerization offers a fast, energy-efficient, and highly controllable route for MIP preparation, particularly suited for micro- and nanoscale architectures. Nonetheless, careful optimization of irradiation conditions and template stability is required to fully exploit its advantages within a green chemistry framework.

#### 3.3.3. Microwave-Assisted Polymerization

Microwave-assisted polymerization has been increasingly explored as a low-energy and time-efficient strategy for MIP synthesis. Unlike conventional thermal heating, which relies on heat transfer from the vessel walls to the reaction medium, microwave irradiation induces rapid and uniform volumetric heating through direct interaction with polar molecules, ionic species, and dipoles present in the reaction mixture. This mechanism enables fast temperature ramping and improved energy efficiency, significantly shortening polymerization times from several hours to minutes [111].

In the context of molecular imprinting, microwave-assisted synthesis has been shown to promote homogeneous nucleation and more uniform polymer growth, often resulting in MIPs with improved morphologies, higher surface areas, and enhanced binding-site accessibility. Several studies have reported that microwave irradiation can enhance imprinting efficiency while reducing solvent consumption and reaction time, particularly in bulk and precipitation polymerization systems. Moreover, the rapid heating associated with microwave-assisted processes can help limit side reactions and preserve template–monomer interactions, especially when combined with polar or ionic media such as water, ionic liquids, or deep eutectic solvents [112,113].

From a green chemistry perspective, microwave-assisted polymerization aligns well with the principle of energy efficiency, as it minimizes heat losses and reduces overall energy input. Its compatibility with greener solvents and bio-based components further strengthens its potential for sustainable MIP synthesis. However, despite these advantages, several limitations must be considered. Precise control of reaction temperature can be challenging, particularly in heterogeneous systems, and localized overheating (“hot spots”) may occur if irradiation conditions are not carefully optimized. In addition, the scalability of microwave-assisted MIP synthesis remains limited by reactor design and equipment availability, which may hinder its translation to large-scale production.

Overall, microwave-assisted polymerization represents a promising low-energy alternative to conventional thermal methods, offering significant reductions in synthesis time and energy consumption. Continued optimization of reaction parameters and reactor configurations is required to fully exploit the potential of this approach for the sustainable fabrication of high-performance MIPs.

### 3.4. Template Removal

Following polymerization, the template molecule must be efficiently removed from the polymer network to generate accessible and selective binding cavities. Template removal is a critical step in MIP synthesis, as incomplete extraction directly reduces the number of available recognition sites and compromises rebinding capacity and extraction efficiency. Moreover, harsh removal conditions—such as extreme pH values, elevated temperatures, or excessive solvent exposure—may induce polymer swelling or cavity deformation, thereby reducing selectivity and reproducibility.

Traditionally, template removal is achieved through exhaustive solvent extraction, most commonly via Soxhlet extraction with large volumes of organic solvent. Although effective, this approach is time-consuming, often requiring several hours to days, and involves substantial consumption of frequently hazardous solvents, making it poorly aligned with green chemistry principles. In addition to its environmental burden, prolonged solvent exposure may promote template degradation or damage to the imprinted polymer structure [114,115].

To overcome these limitations, alternative and more sustainable template-removal strategies have been proposed. Solvent-based approaches include the use of greener extraction media, such as supercritical carbon dioxide and subcritical water, which can significantly reduce solvent toxicity and waste generation. In parallel, physically assisted extraction techniques—such as pressurized liquid extraction, ultrasound-assisted extraction, and microwave-assisted extraction—have attracted increasing interest, as they enhance mass transfer, shorten extraction times, and reduce solvent consumption while preserving the structural integrity of the imprinted cavities [116].

An additional green alternative is electrochemically assisted template removal, which exploits the oxidation or reduction in electroactive template molecules through repeated cyclic voltammetry scans. This strategy enables template elimination without the use of organic solvents and is particularly attractive for MIPs integrated into electrochemical devices. However, its applicability is inherently limited to electroactive templates and to MIPs synthesized on conductive substrates, such as those used in electrochemical sensors [117].

Overall, the choice of template-removal strategy plays a decisive role in determining both the performance and sustainability of MIPs. Continued efforts toward milder, faster, and solvent-efficient extraction methods are essential to minimize environmental impact while preserving imprinting fidelity [118].

## 4. Transduction Method Using New Sensors/Instruments

The miniaturization of analytical devices and the reduction in power consumption have become central paradigms in modern sensing, driven by the growing demand for in situ, real-time, and decentralized analysis. In this context, MIPs have been increasingly integrated into advanced transduction platforms to enable highly selective and sensitive detection while maintaining portability and low energy requirements. The development of MIP-based biochips, nanosensors, optical platforms, and microfluidic devices represents a long-standing and still highly active area of research.

Microfluidic systems, in particular, offer unique advantages over traditional analytical platforms, enabling precise control of fluid handling at the microscale, laminar flow regimes, high-throughput and parallel processing, and rapid analysis with minimal reagent consumption. The incorporation of MIPs into microfluidic architectures allows the fabrication of portable, disposable, and cost-effective sensing devices, well-suited for real-life applications such as environmental monitoring, food safety, and point-of-care diagnostics. These systems benefit from short diffusion paths and enhanced mass transfer, thereby improving response times and analytical performance [119].

Electrochemical transduction remains one of the most widely adopted approaches for MIP-based sensing due to its simplicity, sensitivity, and compatibility with miniaturization. In this regard, screen-printed electrodes enable in situ electrochemical analysis with compact, inexpensive instrumentation, thereby facilitating the development of portable, user-friendly MIP-based sensors [120,121,122]. Similarly, optical transduction platforms, such as MIP-modified optical fibers, have been explored for rapid, remote analysis, offering advantages including real-time detection and resistance to electromagnetic interference [123,124,125].

Among emerging sensing platforms, smartphones have attracted considerable attention as versatile and ubiquitous analytical tools. Modern smartphones integrate high-resolution cameras, powerful processors, multiple sensors, large-capacity batteries, and wireless communication capabilities, making them well-suited for on-site quantitative analysis. Compared with conventional laboratory instruments, smartphone-based sensing platforms offer several advantages, including low cost, portability, rapid data acquisition and processing, wireless data transmission, and ease of operation for non-specialist users. These features make smartphone-assisted MIP sensors particularly attractive for applications in resource-limited settings and remote locations [126,127].

Despite these promising developments, some limitations remain. The sensitivity, accuracy, and long-term reproducibility of certain miniaturized and portable devices may still fall short of those achieved with centralized laboratory instrumentation. Nevertheless, continuous improvements in sensor design, signal processing, and device integration are expected to gradually close this performance gap. With ongoing advances in wearable electronics, microfabrication, and low-power transduction technologies, MIP-based miniaturized sensing platforms are poised to become robust and sustainable alternatives to traditional analytical systems [128,129].

## 5. Conclusions and Perspectives

In conclusion, fourteen guiding principles have been proposed to frame the concept of green molecular imprinting technology (MIT), and the main advances toward its sustainable implementation have been critically assessed. The *greenification* of MIT has primarily progressed along three major directions: reducing health risks associated with exposure to chemical and biochemical reagents, mitigating the environmental impact of conventional imprinting practices, and expanding the applicability of MIPs in areas such as biomedicine, catalysis, tissue engineering, and environmental remediation through the use of safer, biodegradable, and eco-compatible materials.

Despite notable progress, several challenges and opportunities remain. First, although computational modeling has provided valuable insights into imprinting mechanisms, current methods are still insufficient to fully capture the complexity of imprinting processes. The development of faster and more accurate computational tools that minimize experimental trial-and-error is therefore highly desirable. Second, greater emphasis should be placed on the design and application of bio-based and renewable materials in MIP synthesis. Substituting conventional reagents with bio-derived monomers, surfactants, and additives is a promising strategy to reduce emissions and improve sustainability throughout the MIP life cycle.

Third, further advances in aqueous, solvent-free, and low-solvent imprinting strategies are essential. Fourth, the limited durability of flexible polymeric MIPs remains a concern, as deformation of binding sites during repeated use may lead to the premature disposal of the material. The incorporation of imprinting concepts into robust, non-toxic inorganic or metal-based matrices may provide a viable solution by enhancing mechanical stability and reusability.

Equally important is the lack of systematic knowledge regarding the environmental fate of spent MIPs. Issues related to degradation, persistence, and the release of hazardous captured templates require urgent attention. Safe deactivation protocols before disposal, as well as recycling strategies for recoverable components such as porous supports, are critical for minimizing environmental risks and enabling the circular use of materials.

**Figure 10:**
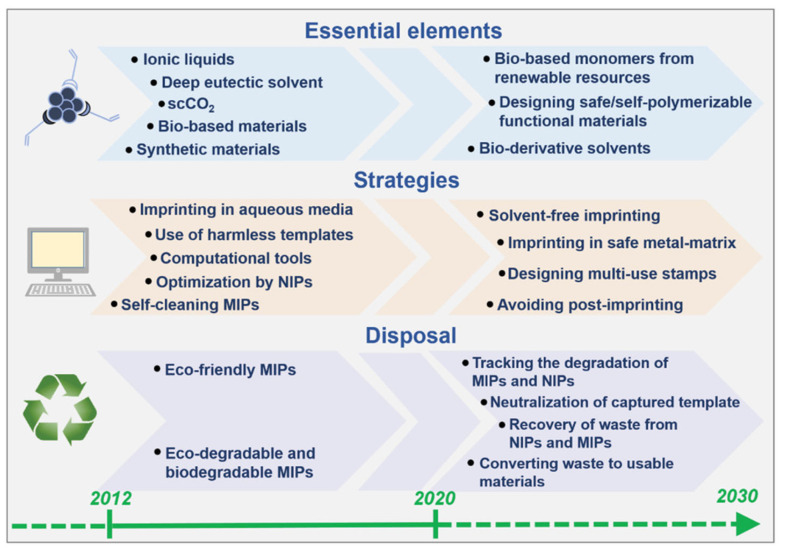
Roadmap to guide the development of green MIT. Reprinted with permission of [21]. Copyright 2021, John Wiley and Sons.

Regardless of their design, a comprehensive life-cycle assessment—from raw materials through to end of life—is necessary to accurately evaluate the sustainability of MIPs. To this end, a strategic roadmap has been proposed to guide the development of green MIT over the coming decades (see Figure 10). While regulatory frameworks and economic incentives can accelerate the adoption of greener practices, researchers have a fundamental responsibility to actively promote sustainable solutions.

In conclusion, greener approaches in MIP synthesis should not be regarded as absolute substitutes for conventional methodologies, but rather as incremental and application-driven improvements aimed at reducing environmental impact without compromising functionality. The nature of the template molecule, the intended application, and a realistic evaluation of sustainability trade-offs must guide the selection of solvents, monomers, and polymerization strategies. In this context, hybrid strategies such as the partial replacement of conventional solvents, reduction in solvent volumes, adoption of low-energy polymerization techniques, or use of greener components when compatible represent practical and effective pathways toward achieving more sustainable molecular imprinting while preserving the recognition performance of MIPs.

## Data Availability

No new data were created or analyzed in this study. Data sharing is not applicable to this article.
